# Cardiovascular Diseases in Central and Eastern Europe: A Call for More Surveillance and Evidence-Based Health Promotion

**DOI:** 10.5334/aogh.2713

**Published:** 2020-02-26

**Authors:** Narine K. Movsisyan, Manlio Vinciguerra, Jose R. Medina-Inojosa, Francisco Lopez-Jimenez

**Affiliations:** 1 International Clinical Research Center, St. Anne’s University Hospital, Brno, CR; 2 Division of Preventive Cardiology, Department of Cardiovascular Medicine, Mayo Clinic, Rochester, Minnesota, US

## Abstract

**Objectives:**

The paper aims to identify the priorities for cardiovascular health promotion research in Central and Eastern Europe (CEE), the region with the highest cardiovascular diseases (CVD) burden in the world.

**Methods:**

This narrative review covered peer-reviewed publications and online databases using a nonsystematic purposive approach.

**Results:**

In despite of a steady decrease in CVD burden in the region, the East-West disparities are still significant. There is minimal continuity in the past and current CVD prevention efforts in the region. Many challenges still exist, including an opportunity gap in research funding, surveillance and population-based preventive interventions. A comprehensive approach focusing on multisectoral cooperation, quality and accessibility of healthcare and equity-oriented public policies and supported by well-designed epidemiologic studies is needed to overcome these challenges.

**Conclusion:**

The current level of effort is not adequate to address the magnitude of the CVD epidemic in CEE. It is imperative to strengthen the epidemiological base concerning cardiovascular health in the region, to foster surveillance and progress in implementation of CVD preventive strategies in the most affected populations of Europe.

## Introduction: Epidemiologic Background and Trajectory

Cardiovascular diseases (CVD) remain the leading cause of death in Europe accounting for 45% of all deaths. More than 4 million Europeans die every year from CVD, primarily from coronary heart disease (CHD) and stroke. Many more are hospitalized and many develop long term disability and require lifelong treatment [1]. Besides the human suffering, CVD has major economic implications for Europe. The economic cost CVD imposes to the EU economy is estimated at €210 billion a year, which includes direct health care costs and non-health costs such as productivity losses and the informal care of people with CVD [2]. The region including Central and Eastern Europe (CEE/CIS) has the highest cardiovascular disease mortality in the world [3,4].

Although CVD mortality in Europe has declined over past decades, the pace of this decline in the European region has significant variability [5]. The World Health Organization’s (WHO) European region includes 53 states with diverse sociopolitical and economic backgrounds. In this article we refer to two groups of countries: 1) CEE, including eleven ex-socialist countries (Bulgaria, Croatia, the Czech Republic, Estonia, Latvia, Lithuania, Hungary, Poland, Romania, Slovenia, and the Slovak Republic) joining EU since 2004, and 2) CIS, the Commonwealth of Independent States of the formerly soviet nations (Armenia, Azerbaijan, Belarus, Georgia, Kazakhstan, Kyrgyzstan, Moldova, Russian Federation, Tajikistan, Turkmenistan, Ukraine, and Uzbekistan).

The CEE countries have the highest CVD mortality in the EU For example, the age-standardized CVD death rates (ASDR) in Latvia and Romania are twice as high than the EU average (883 and 951 vs. 373.6 per 100 000 inhabitants, respectively) [1,6]. Some EU countries such as France and Spain but also outside EU such as Israel have strikingly lower ASDRs (275.2, 292.4, and 255.0, respectively) demonstrating a wide gap when compared to CEE countries.

Not only the CVD death rates are higher in CEE and CIS, but they also occur at younger ages. The premature mortality from CVD (<65yrs) among men in Russia and Belarus is more than ten times higher than in Switzerland (300 vs. 26 per 100 000, respectively). The CVD mortality for men aged 55–59 years in some CIS countries (Belarus, Kazakhstan, Kyrgyzstan, Russia and Ukraine) is higher than for men in ages of 75–79 years in France. Among women, when comparing CEE to other countries in Europe, the premature mortality shows similar patterns as for men though at lower rates [1,2]. The dramatic East-West gap in CVD mortality has emerged with the rapid decline in CVD mortality in Western Europe [7]. This remarkable decline has been explained by combined effects of lifestyle changes, public policy and new, more effective medical treatments for CVD [4,8,9]. To quantify the relative contribution of the determinants of this decline, the IMPACT coronary heart disease model developed by Capewell et al accounts for major CHD risk factors and all established medical interventions for CHD [10]. Though the relative contribution of preventive strategies and medical interventions may vary from country to country, the IMPACT model suggested that more than half the decline in the number of CHD deaths may be attributable to reductions in major risk factors and less than half to evidence-based medical therapies [11,12,13].

**Figure 1:**
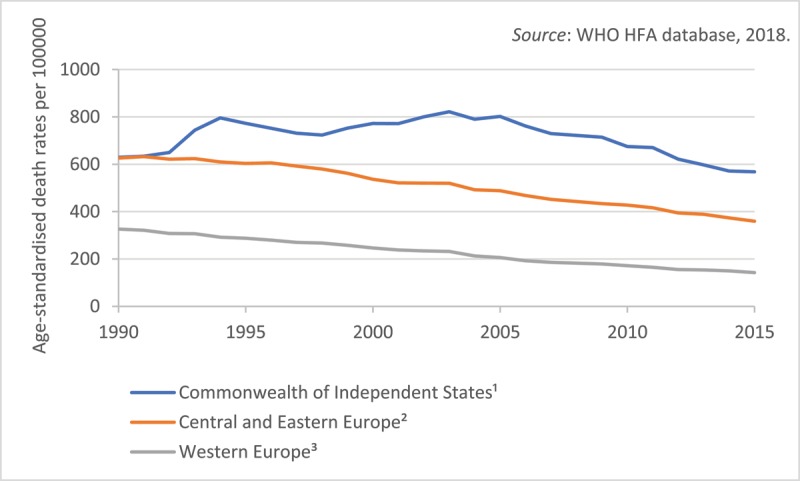
Mortality from diseases of circulatory system in European region by group of countries, 1990–2015. *Notes*: ^1^ Includes Armenia, Belarus, Georgia, Kazakhstan, Kyrgyzstan, Moldova, Russia, Turkmenistan, Ukraine, Uzbekistan. ^2^ Includes Bulgaria, Croatia, the Czech Republic, Estonia, Latvia, Lithuania, Hungary, Poland, Romania, Slovenia, and the Slovak Republic. ^3^ Includes Austria, Belgium, Denmark, Finland, France, Germany, Greece, Ireland, Italy, Luxembourg, Netherlands, Portugal, Spain, Sweden, UK.

Following the eastern block’s collapse in early 1990s, the CVD mortality slowly decreased also in the EU new states, first in Slovenia, Czech Republic, and Poland [7,9]. Studies suggested that similar to Western Europe, more than half the CHD mortality decline in the Czech Republic and Poland can be explained by changes in lifestyle factors. New medical treatments were accountable for 43% and 37% of the decline in these CEE countries, respectively [14,15]. In sharp contrast, CVD and total mortality dramatically increased in the CIS countries, to be improved only by 2010 [16]. Moreover, significant disparities were present by 2015 between CIS and CEE countries, with age-standardized CHD mortality rates (per 100 000 population) ranging from 582.5 in Ukraine to 78.4 in Slovenia, according to WHO Health for All Database 2018 (Table 1). Using the Global Burden of Disease Study 2015 methodology, Murthy et al suggested almost a two-fold gap in CHD burden between CIS and CEE countries [17]. Despite the progress reducing CVD mortality in the region, the East-West disparities persist, with the CEE/CIS lagging behind the west (Figure 1).

**Table 1:** Age-standardized death rates from ischemic heart disease (per 100,000 population) in the countries of Central and Eastern Europe and Commonwealth of Independent States by country and by sex.

Country	Latest year	Males		Females		All	

		Age-standardized mortality, all ages	10-year change in mortality rate (%)	Age-standardized mortality, all ages	10-year change in mortality rate (%)	Age-standardized mortality, all ages	10-year change in mortality rate (%)

Armenia*	2016	337.9	N/A	206.1	N/A	263.2	N/A
Belarus*^,†^	2015	571.4	*–9.5*	255.6	*–16.8*	371.4	*–12.6*
Bulgaria	2014	140.9	*–37.5*	72.0	*–43.6*	102.4	*–40.3*
Croatia	2016	171.9	*–17.5*	101.0	*–18.5*	132.6	*–17.3*
Czech Republic	2016	177.1	*–20.7*	97.9	*–24.2*	132.1	*–21.8*
Estonia	2015	184.7	*–51.8*	88.3	*–53.6*	125.3	*–52.0*
Georgia*	2015	134.2	*–40.6*	73.0	*–35.2*	98.3	*–36.7*
Hungary	2016	246.7	*–22.7*	139.2	*–24.9*	183.7	*–23.7*
Kazakhstan*	2015	152.0	*–70.8*	73.3	*–74.4*	105.1	*–72.4*
Kyrgyzstan*	2015	511.4	*7.5*	356.0	*9.0*	421.9	*7.5*
Latvia	2015	316.1	*–29.8*	156.0	*–23.6*	216.8	*–26.6*
Lithuania	2016	380.0	*–18.3*	196.1	*–22.2*	267.3	*–20.6*
Moldova*	2016	435.9	*–31.7*	292.6	*–34.7*	353.0	*–32.6*
Poland	2015	100.1	*–38.8*	44.9	*–42.8*	68.6	*–40.1*
Romania	2016	198.0	*–26.3*	115.5	*–31.7*	152.2	*–28.7*
Russia*	2013	413.0	*–31.3*	210.6	*–28.2*	289.1	*–29.9*
Slovakia	2014	241.3	*–31.5*	148.4	*–33.5*	187.4	*–32.1*
Slovenia	2014	78.4	*–31.9*	32.4	*–44.5*	52.5	*–36.3*
Turkmenistan*	2013	365.7	*–35.5*	240.3	*–37.4*	295.1	*–36.4*
Ukraine*	2015	582.5	*–21.0*	340.0	*–19.9*	433.1	*–20.3*
Uzbekistan*	2014	371.7	*–13.7*	257.6	*–17.4*	309.3	*–15.6*
Western Europe**	2015	73.8	*–33.9*	33.0	*–39.2*	51.2	*–35.5*
WHO European region***	2015	193.2	*–28.4*	106.9	*–26.0*	142.9	*–27.1*

Notes: NA – not available. * Commonwealth of Independent States membership, either former or current. Recent data not available for Azerbaijan and Tajikistan. ^†^ Change in rates for Belarus is over 11 years, due to missing data. ** Average mortality rates for the Western European countries. *** Average mortality rates for the WHO Euro region. Source: WHO Health for All Database, 2018.

Improving cardiovascular health in population can be challenging but remarkable stories of CVD reduction projects in several countries of the world have proven that this is difficult but not impossible. Those efforts, such as the North Karelia project in Finland, prove that the CVD epidemic can be reversed and that the change can occur in a relatively brief period of time [18].

In this paper, we summarize historical and contemporary approaches in cardiovascular health promotion and suggest research priorities relevant to public health professionals in CEE/CIS. In doing so we hope to assist the efforts to promote and strengthen cardiovascular health in populations in Europe afflicted by the CVD epidemic. Importantly, such efforts need to be effectively executed against constrained resources, competing priorities and competing interests, both regionally and nationally.

## Methods

### Literature search

We identified published papers from academic databases, mainly MEDLINE, through PubMed search (http://www.ncbi.nlm.nih.gov/pubmed) using keywords such as “Central and Eastern Europe”, “Eastern Europe”, “former Soviet Union”, “CVD mortality”, “cardiovascular health”, “cardiovascular prevention”, “cardiovascular health promotion”, “CVD prevention”, “population-based”, “community-based”, and their combinations. Data on CVD mortality and risk factors prevalence were extracted from the WHO Health For All Database and the Eurostat website. We also searched the ClinicalTrials.gov website for any relevant epidemiological studies in the region. Additionally, a hand search using references in identified articles was performed.

## Results: Key Concepts

### CVD risk factors

The traditional or major CVD risk factors identified in the Framingham Heart Study include age, sex, blood lipids, high blood pressure, diabetes, obesity and smoking, along with primordial risk factors such as physical inactivity and unhealthy diet. Those factors have been expanded later by a number of behavioral, psychosocial, environmental and socioeconomic factors, including stress, depression, limited social support and air pollution, among others [19]. Extensive epidemiologic studies have identified novel risk factors such as clinical and subclinical systemic inflammation, microalbuminuria, elevated lipoprotein A and prothrombotic factors. However, the traditional risk factors could explain more than 80% of the excess risk for CHD at the population level [19]. Models for CVD risk prediction can identify persons in need of more aggressive preventive strategies and clinical interventions. Most of the major risk factors can be prevented or reduced through individual and population-based health promotion strategies [20].

**Figure 2:**
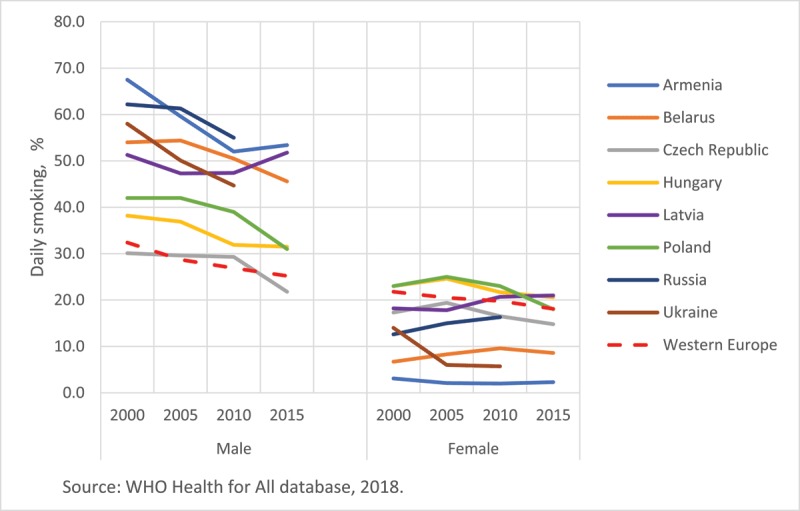
Changes in prevalence of daily smoking in selected countries in Europe, by sex, 2000–2015. Notes: Daily smoking in persons ≥15 years, 2000–2015. Data were not available for Russia and Ukraine after 2010.

**Figure 3:**
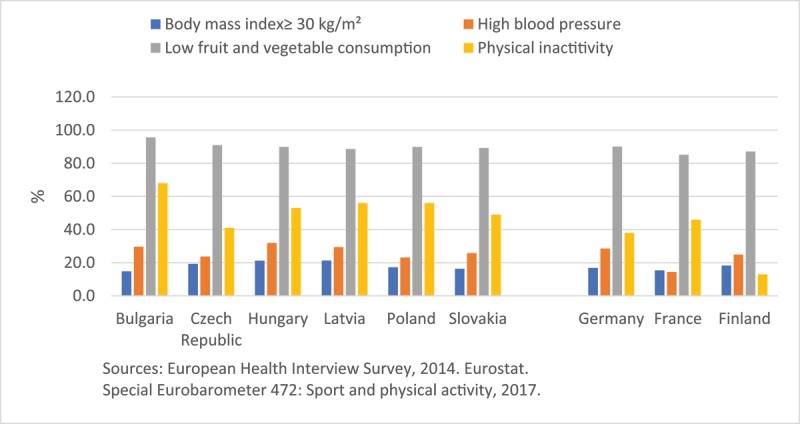
Prevalence of obesity, high blood pressure, low fruit and vegetable consumption and physical inactivity in selected countries in Europe. Notes: Obesity defined as a body mass index (BMI) ≥30 kg/m². High blood pressure: self-reported high blood pressure in the past 12 months. Low fruit and vegetable consumption: less than the daily recommendation of 5 servings of fruits and vegetables. Physical inactivity: never exercising or playing sport.

As expected, the CEE/CIS region has a high prevalence of cardiovascular risk factors, including some of the highest rates of male smoking (Figure 2) and heavy alcohol consumption in Europe and the world [21]. Hypertension and unhealthy diet are major contributors to CVD burden in the CEE/CIS and also in Europe [1]. The CEE/CIS has the highest ASDRs attributable to combined effects of high levels of blood pressure, cholesterol, plasma glucose and body mass index, in the world [22]. Hypercholesterolemia has decreased in the European region as a whole but not in the CEE/CIS where diabetes mellitus and obesity (men) have been on the rise [22]. A recent systematic review found consistently lower fruit intake in CEE/CIS populations compared to Western Europe, but no consistent difference for vegetable intake [23]. There are gaps in monitoring physical activity levels in the region; however, physical exercise seems to be less prevalent in the CEE countries (Figure 3). A decrease in physical activity among children was noted. It also appears that educational level modulates physical activity in different directions. Those with lower education reported higher physical activity in Bulgaria and Romania while in the Czech Republic and Slovakia, conversely, more educated persons were more physically active [24]. Although moderate alcohol consumption has been associated with reduced CVD rates, excessive alcohol consumption can increase CVD rates. Heavy alcohol consumption has been on rise in many CEE countries varying from 25.8% in Bulgaria to 59.4% in the Czech Republic among men and from 4.0% in Estonia to 16.0% in Hungary among women [21]. Certainly, alcohol consumption alone could not explain the excessive CVD burden in CEE [17,25].

### The concept of ideal cardiovascular health

The vision of prevention as merely the absence of disease has been transformed toward a concept of positive health in a paradigm shift described as “a quiet revolution” [26]. Positive health emphasizes an optimal state of health that is more than just an absence of physical, mental or emotional illness: the health is an asset, a resource for wellbeing and thriving. As such, it goes beyond preventive care and includes what is called primordial prevention, which is the prevention of risk factors through lifestyle modification before they occur, as we explain in more detail later in this article. Positive health corresponds closely to the concept of ideal cardiovascular health brought forward by the American Heart Association (AHA) [27]. In line with the vision of positive health, the optimal combination of seven healthy behaviors and health factors, in the absence of CVD, determines the ideal CV health. To meet the definition of ideal cardiovascular health, a person would need to have all 7 metrics at the ideal level, i.e. no smoking within the last year, physical activity at goal, healthy diet conducive to heart health, untreated total cholesterol <200 mg/dL, untreated blood pressure <120/80 mm Hg, BMI < 25kg/m², and untreated fasting plasma glucose <100 mg/dL. Though not without limitations, the concept of ideal cardiovascular health can be used for evaluating and setting strategic goals [28,29].

### Primordial vs. remedial strategies in cardiovascular health promotion

Labarthe makes a clear distinction between CV health promotion and CVD prevention by contrasting their strategies as aligned with different goals [30]. CVD prevention is tasked with reducing rates of CVD risk factors to prevent CVD across several levels of clinical prevention, from the primary (addressing risk factors in persons at risk), the secondary (deterring the disease recurrence) and the tertiary (preventing disability and death) prevention, to population-based prevention. The clinical (high-risk) and population-based (low-risk) approaches are aligned with goals of reducing already established CVD risks. These strategies are coherent with the key concept of CVD prevention that a small shift in the risk of disease across the whole population can lead to a greater reduction in the disease burden than a larger shift in a smaller group of high-risk population [31]. These strategies are remedial, in contrast to primordial strategies aligned with the goals of health promotion, that is the prevention of the CVD risk factors in the first place [30,32]. Interestingly, behavioral interventions like promoting physical activity and a healthy diet aimed to improve the control of risk factors like diabetes, obesity, hypertension and dyslipidemia will also have a powerful effect as primordial preventive strategies. Those behavioral interventions can *prevent* the very same conditions they are intended to *control*. Because the accumulation of risk factors may occur already in childhood, the preventive efforts need to be implemented over a life course, as early as in infancy and earlier.

### Population-wide CV health promotion strategies

Unlike clinical interventions focusing on individual health, population-based preventive strategies target the whole population or entire segments of the population or settings such as workplaces, schools or public policies affecting at the local, national and international level. These strategies are based on the recognition of societal factors in shaping health, or socioeconomic determinants of health [33]. Such strategies may include smoke-free policies, marketing bans, built environment to facilitate healthier lifestyles (e.g., commuting infrastructure, cycling paths), fiscal measures (taxation on unhealthy food products), and other “best buys”. The “best buys” refer to cost-effective or low-cost interventions recommended by the WHO for the dissemination across different settings and populations to reduce non-communicable disease [34]. Thus, the first global public health treaty, the WHO Framework Convention for Tobacco Control, was a success story that facilitated a decrease in smoking rates in many countries including the CEE [35]. Other strategic initiatives include the EU Directives on Tobacco Products, the EU Initiative on Obesity, WHO Global Strategy to Reduce the Harmful Use of Alcohol, and the WHO Global Strategy on Diet, Physical Activity, and Health. Recognized as a major threat to sustainable development, non-communicable diseases (NCDs) are now in the UN agenda, with the goal to reduce premature mortality from NCDs by a third by 2030 [36].

### Cardiovascular community-based prevention: lessons learned

In the past, the CVD prevention goals were often elusive. Early community-based prevention projects, even when sufficiently funded, showed only modest effects on cardiovascular mortality and morbidity, with a few exceptions [37]. The limited efficacy of such interventions was explained by methodological issues, a low exposure to preventive interventions and secular trends [38]. Perhaps the most widely known and cited population-based intervention to reduce CVD occurred in North Karelia where CHD mortality decreased from 1970s to 1995 by 73% following reductions in smoking, hypertension and intake of saturated fats [18,39]. Building on this success, the WHO launched the Countrywide Integrated Non-Communicable Disease Intervention (CINDI) Programme. One of the important lessons of the CINDI Program was that the sustainability of large-scale preventive interventions depends not only on the available resources but also on the political will to mobilize them [40].

Recently, the AHA suggested a comprehensive cardiovascular health promotion model to improve cardiovascular health in community settings [41]. This model embraces surveillance, education and media, partnerships, and availability of and access to health services along with environmental and policy changes. Though the AHA model underscores the community participation and engagement, it lacks specific recommendations to address health inequalities in disadvantaged communities.

### Monitoring the CVD epidemics in Europe: the WHO MONICA Project

The WHO Multinational Monitoring of Trends and Determinants in Cardiovascular Disease (MONICA) Project was launched in 1980s to study 10-year trends in CVD on mortality, morbidity and risk factors across 38 populations in 21 countries [8,39]. The study revealed large differences in CVD outcomes across the settings. In all populations, men had significantly higher all-cause and coronary mortality rates than women. In most populations, the annual CHD incidence declined over time, with Finland reporting the largest reduction in men (6.5% per year over 10 years period) [39,42]. This trend was confronted by increased events among men in Eastern European cohorts, from Russia, Poland, Lithuania, and then-Yugoslavia, but also in China, Catalonia, and Belgium. In most settings, smoking in men, systolic blood pressure and blood cholesterol in both sexes declined. However, the decreasing trend in daily smoking among most of Eastern European men was not significant except for Poland and the Czech Republic. Female smoking increased in most European sites, with greatest increase in southern and eastern European populations such as Spain, Poland, and Russia, where the prevalence was previously low [42]. Serum cholesterol and systolic blood pressure trends were modestly downward in most populations. BMI increased in western populations while it decreased in the East. These diverge trends indicate a persistent need in monitoring the CVD risk factors across European regions.

### Challenges and opportunities for CV health promotion in CEE/CIS

The divergent trajectories since the 1990s have greatly influenced population health trends in the region. The EU new states had the advantage to bring their health policies and health information systems in line with the EU regulations and requirements [43]. They also had opportunities to advance healthcare research and practices using EU and other funds, and to be exposed to EU-wide health awareness campaigns. Nevertheless, public health remained underfunded in the region through a course of prolonged healthcare reform [44]. The concept of evidence-based public health was introduced to the region through the efforts of the Open Society Foundations and the Association of Schools of Public Health in the European Region aimed at capacity building and technical assistance [45,46]. Two decades later many challenges remain. Cardiovascular health continues to compete with other priorities such as maternal and child health and communicable diseases in some countries while in others national health priorities may be sensitive to the agenda of large donor organizations. The still strong influence of multinational tobacco, food and alcohol corporations represents another barrier to effective preventive policies in the region. The tobacco industry interference with public policies has been documented around the globe and the CEE are not the exception [47].

Large social disparities have shown to increase CVD mortality. The socioeconomic restructure that occurred after the fall of communism led to major income gaps and has likely contributed to the high CVD burden in the region [9]. Furthermore, an inadequate government health expenditure in CIS countries (less than 5% of GDP) is a barrier to clinical prevention (e.g., hypertension control), neither it affords the protection against catastrophic expenditures in case of acute CVD events. In additions, several of the CEE/CIS countries contain large rural areas where the life conditions and the access to contemporary treatments and technologies can significantly differ from that in metropolitan areas [48].

Our understanding of previous efforts to prevent CVD in the region is limited by the lack of publications or public data [45]. In countries such as Slovenia and Lithuania, the governments took an ownership of the health promotion infrastructure, while in Poland it was the civil society that mobilized resources to fight against tobacco use [49]. More recently, several nationwide prevention programs have started in the Russian Federation, Kazakhstan and in Belarus [50]. However, the effectiveness of these programs in improving cardiovascular health outcomes on the longer-term is yet to be established [51].

### Cardiovascular surveillance and epidemiologic research in the CEE/CIS

In the post-MONICA period, standardized health data have been collected in the EU through the European Health Interview and the European Health Examination surveys. However, small national samples in both surveys limit the comparability of findings [52]. On a positive note, the participation in population-based CVD registries has increased; most CEE/CIS countries are currently involved in one or more specialized registries of the European Society of Cardiology. Recently, the Enhancing and Accelerating Stroke Treatment (ESO-EAST) collaborative project was launched to improve the stroke outcomes particularly in the CEE [53]. Other initiatives such as EUROASPIRE have garnered participation from the professional societies in the CEE [54]. Those surveys help to track changes in the management of patients with established CVD but unfortunately do not provide information regarding incidence and mortality rates of CVD or trends in prevalence of CVD risk factors. There are only a few prospective population-based cohort studies to investigate those trends and to assess mediators and outcomes in these populations that are most affected by CVD. A few notable exceptions are described below.

The Health, Alcohol and Psychosocial factors In Eastern Europe (HAPIEE) is a large prospective cohort study in Russia, Poland, the Czech Republic and Lithuania with about 36,000 participants [55]. The study confirmed the role of conventional risk factors and also reported the associations between socioeconomic status, nutrition, alcohol, social support, depression and CVD mortality.[21,56] These findings, however, could not explain the excess CVD mortality in Russia compared to Poland and the Czech Republic [55].

The Prospective Urban Rural Epidemiology Study (PURE) is an on ongoing large-scale project with 34 sites, including four in the CEE/CIS countries [57]. The recent findings suggested that physical activity, both recreational and non-recreational, were associated with reduced CVD events whereas diets high in carbohydrates were associated with higher total mortality, possibly due to low variety of available foods [58].

The Kardiovize Brno 2030 is a prospective cardiovascular cohort of 2160 residents of Brno, the second city in the Czech Republic [59]. While building on the Czech post-MONICA survey, the Kardiovize examines a broader range of CVD risk factors, including cardio-ankle vascular index, intima media thickness, body fat distribution, and genetic factors [60]. This study has recently confirmed that the baseline prevalence of ideal or near ideal cardiovascular health in the study population was low (19.1%) [61]. About 66.0% of the participants had hypertension, 68.8% had high cholesterol and less than 27% consumed fruit and vegetables on a daily basis. These preliminary findings underscore the need for a special focus on healthier food choices.

## Discussion: Unmet needs and future directions

The cardiovascular health promotion research area remains largely underdeveloped in CEE [62]. There is a regional bias across the EU with the research funding favoring western countries for whom many domestic sources exist whereas the CEE countries have the highest CVD burden and comparatively low research efforts [63]. The disparities in research funding can be characterized as a two-tier opportunity gap, due to the lack of funding and the lack of capacity for participation.

The European region is considered “a natural epidemiologic laboratory” that, due to its enormous diversity, can provide lessons to everyone [16]. Yet, adapting known effective interventions may not be sufficient to overcome the CVD burden in the CEE/CIS, a region with remarkably differed mortality and risk factor prevalence trends and with paucity of data [64]. New, innovative solutions must be sought to improve the cardiovascular health in the region. When adapted, these efforts should take into consideration the local context, idiosyncrasy, traditions, social factors and also equity implications [65]. A comprehensive strategy would balance individual and population-based approaches through multi-sectoral interventions targeting both healthcare systems and societal roots of the disease [64]. Such comprehensive strategy should emphasize population-based primordial prevention of CV risk factors over a life course, but also consider primary and secondary prevention to reach those at high risk or with established disease, for attaining the greatest possible gains in cardiovascular health [30]. The cost-effective secondary prevention strategies should be prioritized over selected expensive treatments that would benefit a few but would constrain the finances to fund other interventions.

Future efforts should also address the lack of longitudinal epidemiological studies in the region [66]. Such studies could greatly benefit from the participation in European and international cohort consortiums. This may lead to the development of scientific cooperation and cross-fertilization regarding the implementation of preventive strategies.

The persisting East-West CVD mortality gap indicates that the current efforts are insufficient to address this problem. There is an urgent need to increase surveillance of CVD and its risk factors, to improve awareness of the problem among local politicians and the public, to prioritize funding for CVD prevention and to implement population-based interventions to reduce the burden of CVD. Failure to do so will result in unsurmountable healthcare expenses in the short and mid-term that will undermine the already limited economic resources and creating a vicious circle. After all, the most expensive choice for CEE would be *not* spending enough money in CVD prevention.
